# Supplementary material for: SyNDI: synchronous network data integration framework
Source: BMC Bioinformatics. 2018 Nov 6;19:403. doi: 10.1186/s12859-018-2426-5 (PMC6219086; doi:10.1186/s12859-018-2426-5)
Supplement: Supplementary file 5 — Files of the example presented in Additional file 4. This additional file is a zip package that contains all files used in Additional file 4. Detailed descriptions of each of these files are presented in a README file included in this zip package. (GZ 30411 kb) [file 12859_2018_2426_MOESM5_ESM.gz › SyNDITools/Meme2Fimo/meme_4.9.0_4.tar.gz!/meme_4.9.0/website/html/mhmm-papers.in]

Meta-MEME v3.3


> ### Papers & publications
>
> ---
>
> Click on the links below to see abstracts.
>
> - Shobhit Gupta, John A. Stamatoyannopoulos, Timothy L. Bailey and William Stafford Noble.
>   "Quantifying similarity between motifs"
>   *Genome Biology* 8:R24, 2007.
> - Grundy, William Noble. A Bayesian
>   Approach to Motif-based Protein Modeling. Ph.D. dissertation.
>   University of California, San Diego. June, 1998.
> - Grundy, William N., Timothy L. Bailey, Charles P. Elkan and
>   Michael E. Baker. "Meta-MEME:
>   Motif-based Hidden Markov Models of Biological Sequences".
>   *Computer
>   Applications in the Biosciences*, 13(4):397-406,
>   1997.
> - Grundy, William N., Timothy L. Bailey, Charles P. Elkan and
>   Michael E. Baker. "Hidden
>   Markov Model Analysis of Motifs in Steroid Dehydrogenases and their
>   Homologs." *Biochemical and
>   Biophysical Research Communications*, 231(3):760-766,
>   1997.
> - Grundy, William N. and Charles P. Elkan. "Motif-based
>   Hidden Markov Models for Multiple Sequence Alignment" (poster).
>   Fifth
>   International Conference on Intelligent Systems for Molecular
>   Biology (ISMB-97). Halkidiki, Greece. June 21-25,
>   1997.
> - Bailey, Timothy L., Michael E. Baker, Charles P. Elkan and
>   William N. Grundy. "MEME, MAST, and Meta-MEME: New Tools for Motif
>   Discovery in Protein Sequences" in *Pattern Discovery in
>   Molecular Biology.* J. Wang, B. Shapiro and D. Shasha, editors.
>   Oxford UP, 1998. *In press*.
> - Grundy, William N., Timothy L. Bailey and Charles P. Elkan.
>   "ParaMEME:
>   A Parallel Implementation and a Web Interface for a DNA and Protein
>   Motif Discovery Tool" (with corrections). *Computer Applications
>   in the Biosciences* 12(4):303-310,
>   1996.
>
> ---
>
> Return to the Meta-MEME home page.
>
> *Please send comments and questions to:
> @METAMEME\_CONTACT@.*
